# Effect of Horizontal Transfer of Cold-Tolerant Substances of *Ambrosia artemisiifolia* on the Low Temperature Adaptability of *Ophraella communa*

**DOI:** 10.3390/insects17050488

**Published:** 2026-05-10

**Authors:** Xihao Wang, Mengying He, Lu Yang, Yan Zhang, Jingfang Yang, Xueyan Zhang, Zhongshi Zhou

**Affiliations:** 1 Ministry of Education Key Laboratory for Ecology of Tropical Islands, Key Laboratory of Tropical Animal and Plant Ecology of Hainan Province, College of Life Sciences, Hainan Normal University, Haikou 571158, China; wangxihao1999@163.com (X.W.);; 2 National Nanfan Research Institute, Chinese Academy of Agricultural Sciences, Sanya 572024, China; 3 State Key Laboratory for Biology of Plant Diseases and Insect Pests, Institute of Plant Protection, Chinese Academy of Agricultural Sciences, Beijing 100193, China; 4 State Key Laboratory of Resource Insects, Institute of Apicultural Research, Chinese Academy of Agricultural Sciences, Beijing 100193, China

**Keywords:** ragweed, leaf beetle, cryoprotectants, low-temperature adaptability

## Abstract

External environment shapes the cold adaptation of *Ophraella communa*, a specialist natural enemy of the invasive common ragweed, and further affects its biological control efficacy. Our study aims to clarify the cold tolerance correlation between ragweed and leaf beetle and explore whether host cold acclimation can enhance the beetle’s low-temperature adaptability. We measured cold-resistant substances, supercooling point (SCP), and chill-coma recovery time (CCRT) of four geographic populations, and conducted indoor low-temperature acclimation of ragweed at 13 °C, 17 °C, 21 °C for 3–9 d, then fed leaf beetle with treated leaves. Results showed that cold-resistant substances in both species varied with latitude and were highly heritable. Cold acclimation can elevate sugars, trehalose, glycerol, and proline in ragweed; the cold tolerance of beetles feeding on these plants was significantly improved. We conclude that cold-resistant substances in ragweed can be trophically transferred to leaf beetle to enhance its cold hardiness, providing a new strategy to improve biocontrol in high-latitude regions.

## Simple Summary

The ragweed *Ambrosia artemisiifolia* is a destructive invasive weed worldwide, severely threatening agriculture and ecosystems. The leaf beetle *Ophraella communa*, a specific natural enemy, has been verified to effectively control ragweed via herbivory. Releasing this beetle serves as a promising biocontrol strategy. However, while efficient at low and middle latitudes, beetles struggle to establish stable populations in high-latitude regions, thereby failing to exert their biological control effect, which in turn allows the spread of ragweed to remain unchecked. Our study measured cold-resistance substances and indexes of ragweed and beetle populations across latitudinal gradients. We also conducted low-temperature acclimation on ragweed and examined cold tolerance changes in beetles feeding on these plants. After testing beetles fed on 12 groups of treated ragweed under different acclimation temperatures and durations, we confirmed enhanced cold tolerance in the beetles. Previous studies reported that insect cold acclimation improves cold hardiness. Our findings reveal that acclimation of host plants also enhances insect cold tolerance, providing a novel approach for managing other invasive plants.

## 1. Introduction

Biological invasion refers to the process by which non-native organisms (including plants, animals, and microorganisms) spread into new environments -either naturally or through human activities-and cause harm to local biodiversity, ecosystems, or human health [1,2]. With the intensification of human activities, the expansion of economic globalization and the advancement of urbanization, opportunities for the successful colonization of alien species have greatly increased. As a result, the threat to global biodiversity has become increasingly severe, making biological invasion one of the most critical issues in the context of global environmental change [3].

*Ambrosia artemisiifolia*, commonly known as common ragweed, is an annual invasive herb in the family Asteraceae [4]. Originally native to the southern United States and northern Mexico, *A. artemisiifolia* has now spread to more than 30 countries and regions across Europe, America, Asia, and Australia, causing severe harm in many parts of the world [5]. The harmful impacts of *A. artemisiifolia* are mainly reflected in four areas: posing risks to human health, damaging agricultural production, disrupting local plant diversity and serving as an intermediate host for certain plant pathogens and insect pests [6]. Its pollens cause allergic reactions in humans. These reactions manifest as fever-like symptoms such as coughing, sneezing, and fatigue, and can range from mild redness, swelling, and itching to severe chest tightness, shortness of breath, headache, dyspnea, and, in extreme cases, even death [7]. It rapidly outcompetes crops for resources in farmlands and pastures, directly reducing crop yield and quality and causing economic losses. Its vigorous invasion also displaces native plants, lowers local plant diversity, and destabilizes the structure and function of natural ecosystems. Moreover, it acts as an intermediate host for multiple plant pathogens and insect pests, facilitating their spread and worsening associated agricultural and ecological damage [8].

The ragweed leaf beetle *Ophraella communa* is a member of the leaf beetle family Chrysomelidae. Like ragweed, it is native to continental North America. It mainly feeds on plants of the genus *Ambrosia* and exhibits a highly specialized oligophagous feeding habit [9,10,11]. Given its wide distribution across temperate regions, *O. communa* has evolved specific cold-adaptation strategies to cope with seasonal cooling. *O. communa* usually overwinters as mated adults [12]. When daylength falls below 14 h, the leaf beetles stop reproductive development, enter a dormant period, and accumulate a large amount of cryoprotectants to survive the cold season [13,14,15,16,17].

Insects initiate a series of adaptive physiological regulatory responses when entering autumn and winter or exposed to low-temperature stress, which are mainly manifested as significant upregulation in the contents of low-temperature protective substances such as sugars, lipids, and proline in their bodies [18,19]. In addition to intrinsic physiological regulation, host plants can also significantly affect the cold tolerance of insects. The primary underlying mechanism is that different hosts vary in nutritional quality and chemical composition, which directly leads to differences in nutrient acquisition and accumulation after insect feeding, thereby altering the synthesis and accumulation levels of key substances including low-temperature protectants, and ultimately enhancing their tolerance and resistance to low-temperature stress [20].

In this context, we investigated cold tolerance-related indices of ragweed and leaf beetles from different latitudes. We observed that the cold tolerance of *O. communa* populations from relatively higher latitudes is enhanced by the low-temperature conditions of their original habitats. We hypothesized that the enhanced cold tolerance of leaf beetles may be attributed to temperature differences in the original habitats of ragweed. Subsequent verification experiments with different acclimation durations and temperatures were conducted under laboratory conditions.

## 2. Materials and Methods

### 2.1. Wild Samples Collection and Husbandry

The initial study populations consisted of wild *A. artemisiifolia* and *O. communa* collected from Shenyang, Liaoning (41°55′05″ N, 123°42′13″ E), Qinhuangdao, Hebei (39°56′08″ N, 119°26′36″ E), Wuhan, Hubei (31°09′34″ N, 114°34′37″ E), and Nanning, Guangxi (22°51′01″ N, 108°14′45″ E) in China, between late August and early September 2024. During collection, ragweed plants were cut at the stems, and the cut ends were wrapped with moist cotton to ensure plant viability. Ragweed intended for feeding *O. communa* was treated in the same way but kept separate. These individuals were divided into four groups according to their region of origin and labeled accordingly. Leaves were gently wiped with sterile filter paper to remove water and dust, then wrapped in tin foil, immediately frozen in liquid nitrogen, and stored at −80 °C.

The wild-collected *O. communa* were reared indoors on artificially pre-grown ragweed. Ragweed was prepared as follows: before planting, ragweed seeds were soaked in water for 3 days, with the water replaced every 24 h. Seedling trays were then filled with soil, seeds were sowed evenly, and a thin layer was used to cover them. The trays were thoroughly watered immediately after sowing. When the seedlings grew to about 5 cm in height, they were transplanted into nutrient pots. First, nutrient pots were filled with soil up to one-third of their volume. Seedlings were carefully removed from the seedling trays and transplanted into the nutrient pots, ensuring the rhizomes remained upright, after which the soil was gently compacted. After transplanting, the pots were placed in trays and watered thoroughly to ensure full root hydration. Growth conditions were maintained at 25 ± 1 °C, relative humidity of 80 ± 5%, and a photoperiod of L//D = 14 h//10 h. The plants were ready for use after 30 days of growth. After rearing of *O. communa* for one generation, offspring individuals with synchronized eclosion times were collected, placed in centrifuge tubes, immediately frozen in liquid nitrogen, and stored at −80 °C.

### 2.2. Cold-Resistant Substance Testing and Cold-Resistance Test

Sugar, trehalose, glycerol and proline are the main physiological accumulations in plants that adapt to cold stress [21,22], and are widely used to study cold tolerance in plants and insects. Therefore, these four physiological accumulations were chosen for measurement in this study. In addition, water content was determined to indirectly reflect dry matter accumulation.

To determine total sugar content, the test material was crushed in a mortar, and 0.1 g leaf tissue or insect body was weighed and transferred into a 2 mL centrifuge tube. Then, 1.5 mL of distilled water was added, and the mixture was heated at 100 °C for 10 min. After cooling, the sample was centrifuged at 5000 rpm (revolutions per minute) at 25 °C for 10 min. Then, 1 mL of the supernatant was transferred to a 10 mL centrifuge, tube and 4 mL of 0.20% anthrone-sulfuric acid reagent was added and mixed thoroughly. The mixture was heated in a boiling water bath for 10 min, cooled with tap water, and equilibrated for 20 min. A blank tube was used to set the zero point of the solution, and the absorbance at 620 nm was measured and recorded.

To determine trehalose content, the test material was ground in a mortar, and 0.1 g of leaf tissue or insect body was weighed and transferred into a 2 mL centrifuge tube. Then, 1.5 mL of distilled water was added, and the mixture was heated at 100 °C for 10 min. After cooling, the sample was centrifuged at 5000 rpm at 25 °C for 10 min. Subsequently, 1 mL of the supernatant was transferred to a 10 mL centrifuge tube. After adding 0.5 mL of 1.50% H_2_SO_4_ solution, the mixture was heated in a water bath at 90 °C for 10 min. Once cooled, 0.5 mL of 30% KOH solution was added to the mixture, and heating was repeated for another 10 min. Next, 4 mL of 0.20% anthrone-sulfuric acid reagent was added and mixed thoroughly. The mixture was heated in a boiling water bath for 10 min, cooled with tap water, and equilibrated for 20 min. A blank tube was used to set the zero point of the solution, and the absorbance at 620 nm was measured and recorded.

To determine glycerol content, the test material was crushed in a mortar, and 0.1 g leaf tissue or insect body was weighed and transferred to a 2 mL centrifuge tube. Then, 1.5 mL of distilled water was added, and the mixture was heated at 100 °C for 15 min. After cooling, the sample was centrifuged at 5000 rpm and 25 °C for 10 min. Next, 1 mL of the supernatant was transferred to a 10 mL centrifuge tube, followed by the addition of 2 mL of oxidizing reagent (Prepared by dissolving 130 mg of sodium periodate in 50 mL distilled water, adding 8 g anhydrous sodium acetate and 6 mL glacial acetic acid, then diluting to 100 mL with distilled water). Subsequently, 2 mL of color-developing reagent (Prepared by diluting 0.4 mL acetylacetone to 100 mL with isopropanol) was added. The mixture was heated in a 70 °C water bath for 15 min to facilitate color development, cooled under tap water, and equilibrated for 20 min. A blank tube was used to set the zero point of the solution, and absorbance was measured at 420 nm.

To determine proline content, the test material was ground in a mortar, weighed, and then transferred to a 1.5 mL centrifuge tube. 500 μL of sulfosalicylic acid was added to the tube, followed by heating at 100 °C for 10 min. The mixture was then centrifuged at 10,000× *g* and 25 °C for 10 min. After cooling, 0.25 mL of the supernatant, glacial acetic acid, and ninhydrin were separately added to a 2 mL centrifuge tube. The mixture was incubated at 100 °C for 30 min, and shaken every 10 min. After cooling, 0.5 mL of toluene was added, and the mixture was shaken for 30 min to extract proline. Finally, 0.2 mL of the supernatant was selected, and its absorbance was measured at 520 nm using a quartz microcuvette.

To determine the supercooling point (SCP), we randomly selected 100 individuals from four geographical populations. A 2 mm aperture was cut at the tip of a pipette, and the temperature probe was placed at this tip (Shenzhen Shenhwa Technology Co., Ltd., Shenzhen, China). The probe was connected to the insect body, which was then placed in a low-temperature refrigerator set at −20 °C. The insect was cooled at an average rate of approximately 0.5 °C/min. When the body fluids began to freeze, latent heat was released within the insect, generating a distinct curved peak on the recorder. Then, we numbered and documented this peak as SCP.

To compare cold tolerance among populations, 200 individuals were randomly selected from each mass bred population on the third day after eclosion for chill-coma recovery time (CCRT) assessment. Ten adults were randomly chosen from each replicate and placed in centrifuge tubes (one adult/tube), which were then incubated in a temperature-controlled chamber set at −15 ± 0.1 °C for 25 min. After incubation, the centrifuge tubes were removed, dried immediately, and placed on a piece of paper. The adults were allowed to recover at room temperature (25 °C) without any mechanical stimulation. Then, we started a timer to observe the beetles’ activity, recording the CCRT as the period from placement on the paper until the beetle regained an upright position.

All measurements were conducted with a minimum of five replicates. Of all the above reagents, sulfuric acid was obtained from Xilong Scientific Co., Ltd., Shantou, China, and all the other reagents were produced by Shanghai Macklin Biochemical Co., Ltd., Shanghai, China.

### 2.3. Wild Samples Data Analysis

Differences in total sugar, trehalose, glycerol, proline and water content in *A. artemisiifolia* and *O. communa* among populations, as well as differences in SCP and CCRT of *O. communa* among populations, were analyzed using Tukey’s honest significant difference (HSD) test in SPSS 22.0 (IBMCorp., Armonk, NY, USA), and all figures were created using Origin 2024 (OriginLab Corp., Northampton, MA, USA).

Meanwhile, to quantify the relative contributions of environmental factors and genetic effects to phenotypic variation in *A. artemisiifolia* and *O. communa*, we estimated broad-sense heritability (H^2^) for physiological traits across different geographic populations using R 4.5.2 (R Core Team, Vienna, Austria). Broad-sense heritability analysis partitions total phenotypic variance into genetic and environmental components, allowing us to evaluate the degree to which observed trait differences are shaped by genetic background.

### 2.4. Indoor Low-Temperature Acclimation

**Figure 1:**
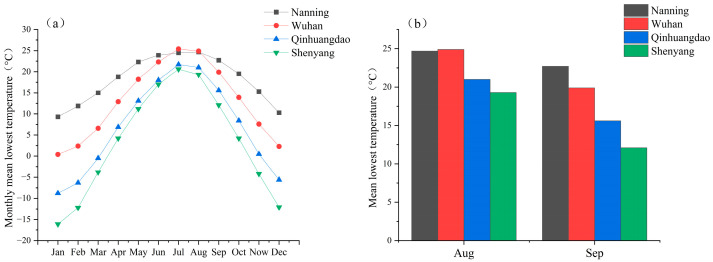
Mean lowest temperature, monthly mean low temperature, and monthly mean temperatures in four regions from 2019 to 2024. Data were obtained from the official website of Weather Atlas at https://www.weather-atlas.com. (**a**) The averages from daily minimum temperature in each month from 2019 to 2024. (**b**) The average monthly minimum temperatures in the autumn months from 2019 to 2024.

According to Weather Atlas|Global Weather Forecasts and Climate Information (https://www.weather-atlas.com), the low-temperature treatment temperatures were set based on the average minimum temperatures in September across four wild sampling regions (Figure 1). To simulate the temperatures of *A. artemisiifolia* before autumn senescence and withering in different latitudinal regions, 13 °C, 17 °C, and 21 °C were selected as the low-temperature experimental groups (13 °C, 17 °C, and 21 °C were designated as Grade III, II, and I, respectively), while 25 °C (normal temperature condition) served as the blank control group. The above *A. artemisiifolia* plants were equally placed in illumination incubators under different temperature treatments, keeping all other conditions consistent with those described in Section 2.1. Meanwhile, to simulate the frequency of autumn cold waves, 3 d, 5 d, 7 d, and 9 d were chosen as the low-temperature treatment durations, with 0 d treatment as the blank control. After the corresponding treatment durations, some *A. artemisiifolia* plants from the experimental groups were taken out for subsequent *O. communa* rearing experiments, while additional 20 randomly picked *A. artemisiifolia* leaves were cooled in liquid nitrogen and stored at −80 °C for subsequent detection of cryoprotectants.

### 2.5. Feeding Treatment for O. communa Adults

The test *O. communa* pupae were collected in March 2025 from the Langfang Experimental Station of the Chinese Academy of Agricultural Sciences (CAAS), Langfang, Hebei Province, China. Upon collection, the pupae were reared on *A. artemisiifolia* plants pre-grown at room temperature. The indoor conditions were the same as those described in Section 2.1. Newly emerged adults of the same cohort were selected and placed in insect-rearing boxes, where they were fed with *A. artemisiifolia* leaves treated at different temperatures and durations for 3 days. The petioles of the leaves were wrapped in moist absorbent cotton to maintain leaf freshness, and the leaves were replaced every 12 h. After continuous feeding for 3 days, the beetles were stored at −80 °C for subsequent detection of physiological accumulations, while live individuals were sampled for the determination of SCP and CCRT.

### 2.6. Indoor Samples Testing and Analysis

The samples of *A. artemisiifolia* and *O. communa* cryopreserved at −80 °C were sampled, ground, weighed, and detected in groups according to their respective low-temperature treatment conditions and durations. The detection methods were consistent with those described in Section 2.2. to determine the contents of total sugar, trehalose, glycerol, water, and proline in both organisms. Meanwhile, we measured the SCP and CCRT of *O. communa* in different treatment groups. All measurements were replicated five times and the test results were also analyzed by HSD test using SPSS to examine the temporal differences within each temperature treatment group. All figures were also created using Origin 2024.

## 3. Results

### 3.1. Differences in Physiological Accumulations Among Four Populations

**Figure 2:**
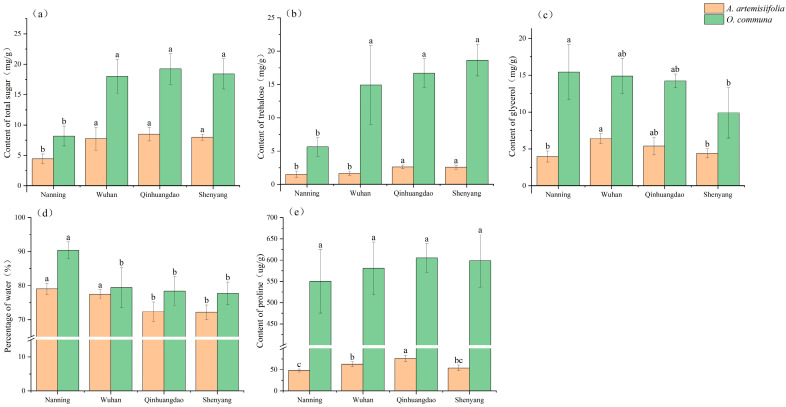
Differences in the physiological content of *A. artemisiifolia* and *O. communa* among four populations. (**a**) total sugar content; (**b**) trehalose content; (**c**) glycerol content; (**d**) water content; (**e**) proline content. Different letters represent significant difference among treatments (*p* < 0.05, Tukey HSD test). The data are shown as mean ± SE.

After quantifying the physiological indices in *A. artemisiifolia* and *O. communa* from different latitudinal populations, the following results were obtained. For total sugar content in *A. artemisiifolia* the Qinhuangdao population exhibited the highest level, and the total sugar content of the Nanning population was significantly lower than that of other populations (*F* = 12.249, *p* < 0.05). Similarly, *O. communa individuals* also exhibited the same pattern of differences as the *A. artemisiifolia* samples (*F* = 11.052, *p* < 0.05) (Figure 2a). For trehalose content, in *A. artemisiifolia*, the high-latitude populations had significantly higher trehalose content than the low-latitude populations (*F* = 14.226, *p* < 0.05). In *O. communa*, trehalose content differed markedly among populations, and showed a numerical increasing trend with latitude (*F* = 14.007, *p* < 0.05) (Figure 2b). Regarding glycerol content in *A. artemisiifolia*, the Wuhan population had significantly higher glycerol content than the other three populations, showing a pattern of mid-latitude elevation and lower values at both the northernmost and southernmost latitudes (*F* = 8.253, *p* < 0.05). In *O. communa*, glycerol content significantly exhibited a decreasing trend with increasing latitude (*F* = 3.917, *p* < 0.05) (Figure 2c). For water content, *A. artemisiifolia* from the two higher-latitude regions was significantly lower than that from the other two regions (*F* = 14.376, *p* < 0.05). In *O. communa*, the Nanning population exhibited the highest water content, which was significantly higher than that of other regions, while no significant differences in water content were observed among the beetle populations from the remaining three regions (*F* = 10.284, *p* < 0.05) (Figure 2d). For proline content in *A. artemisiifolia*, a general increasing trend with latitude was observed. However, the Shenyang population had a lower content than the Qinhuangdao population. Overall, the differences among the populations were significant (*F* = 19.724, *p* < 0.05). Lastly, there were no marked differences in proline content among populations of *O. communa* (*F* = 0.842, *p* = 0.491) (Figure 2e).

**Figure 3:**
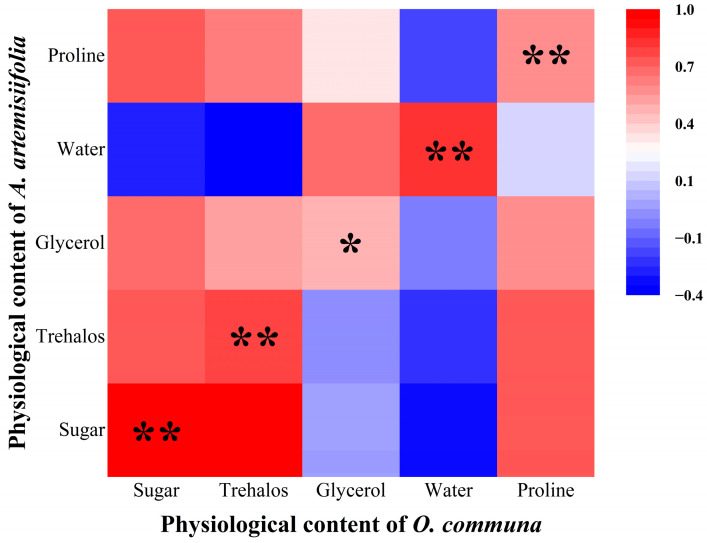
Correlation heatmap of physiological content between *A. artemisiifolia* and *O. communa*. Red represents positive correlation, while blue represents negative correlation; a darker color indicates a stronger correlation. * indicates significant difference (*p* < 0.05), and ** indicates highly significant difference (*p* < 0.01). The horizontal and vertical axes represent the physiological content of *A. artemisiifolia* and *O. communa*, respectively.

To further investigate the correlation between the contents of corresponding physiological substances in *A. artemisiifolia* and *O. communa*, Pearson correlation analysis (all data were tested to conform to a normal distribution; thus, Pearson correlation analysis was employed) was performed to determine the correlation coefficients between their corresponding substances (Figure 3). The results showed that all substances exhibited a significant correlation at the 0.01 level, except for glycerol content, which was only significantly correlated at the 0.05 level. This indicates that cold tolerance-related metabolites in *A. artemisiifolia* are transferred to *O. communa* through feeding and accumulate substantially in the beetles via nutrient transmission.

### 3.2. Differences of Cold Resistance in O. communa Among Four Populations

**Figure 4:**
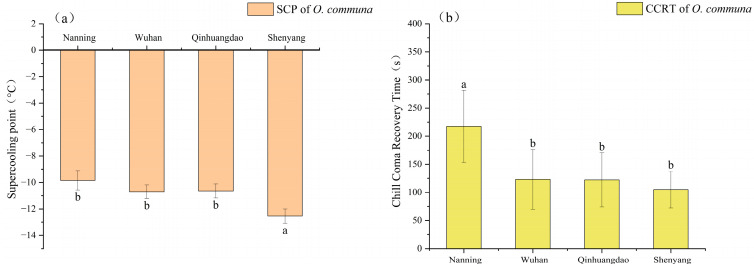
The differences in SCP and CCRT of *O. communa* among four populations. (**a**) supercooling point; (**b**) chill coma recovery time. Different lowercase letters represent significant difference among treatments (*p* < 0.05, Tukey HSD test). The data are shown as mean ± SE.

After assessing the cold tolerance physiological indices of *O. communa* from different latitudinal regions, the following results were obtained. The SCP of the Shenyang population was significantly higher than that of the other three populations (*F* = 19.006, *p* < 0.05) (Figure 4a). In contrast, the CCRT of the Nanning population was significantly lower than that of the other three populations (*F* = 8.086, *p* < 0.05) (Figure 4b). It is inferred that the cold tolerance of *O. communa* tends to increase with the rise in latitude across its populations.

As shown in Table 1, the broad-sense heritability of all indices exceeded 80%. In *A. artemisiifolia*, proline exhibited the highest broad-sense heritability of 98.9%, while glycerol still reached a relatively high value of 97.6%. In contrast, for *O. communa*, the supercooling point showed the maximum broad-sense heritability of 99.0%. Unlike the pattern in *A. artemisiifolia*, proline had the lowest broad-sense heritability of only 80.8% in *O. communa*. These results indicate that the cold tolerance of both *A. artemisiifolia* and *O. communa* is less affected by environmental factors and predominantly governed by genetic effects. In subsequent studies, populations of *O. communa* from high-latitude regions can be selected for progeny breeding, to obtain individuals with enhanced cold tolerance for the biological control of *A. artemisiifolia*.

### 3.3. Differences in Physiological Accumulations in A. artemisiifolia After Acclimation

After *A. artemisiifolia* was acclimated to different temperatures, the following results were obtained regarding its physiological indices compared with those grown at room temperature.

**Figure 5:**
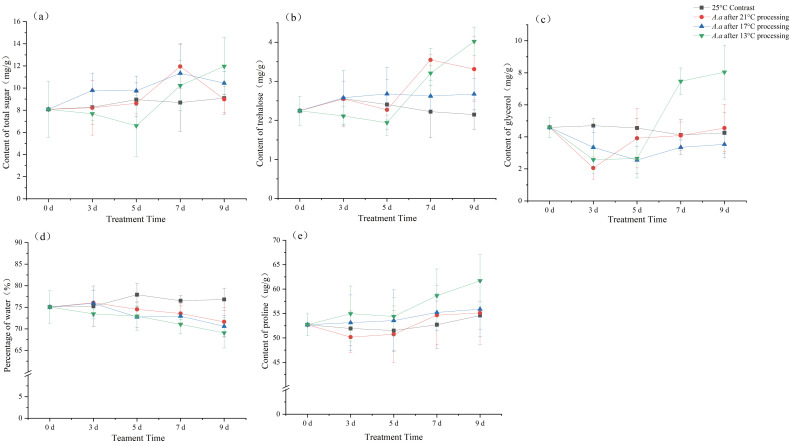
Effect of cold-hardening on the physiological contents of *A. artemisiifolia*. (**a**) total sugar content; (**b**) trehalose content; (**c**) glycerol content; (**d**) water content; (**e**) proline content. The data are showed as mean ± SE.

For total sugar content, no significant difference was observed over time at 17 °C (*F* = 1.901, *p* = 1.5). At 21 °C, significant differences were detected over time (*F* = 3.376, *p* < 0.05), showing a trend of increasing first and then decreasing. The maximum value was obtained after 7 days of acclimation. At 13 °C, the total sugar content exhibited a trend of initially decreasing and then sharply increasing, with the minimum value recorded on the 5th day of acclimation. Significant differences were also found among the sampling days (*F* = 3.951, *p* < 0.05) (Figure 5a). For trehalose content, at 17 °C, there was no significant change over time (*F* = 0.579, *p* = 0.681). At 21 °C, significant differences were observed over time (*F* = 6.652, *p* < 0.05), showing an increasing trend with the maximum value achieved after 7 days of acclimation. At 13 °C, the trehalose content initially decreased and then sharply increased, with the minimum value recorded on the 5th day of acclimation. Significant differences were also detected among the sampling days (*F* = 23.367, *p* < 0.05) (Figure 5b). Glycerol content showed a significant trend of initially decreasing and then increasing in all acclimated groups. At 21 °C (*F* = 3.781, *p* < 0.05) and 13 °C (*F* = 27.358, *p* < 0.05), the minimum glycerol content was reached after 3 days of acclimation. At 17 °C (*F* = 3.686, *p* < 0.05), the minimum value was observed on the 5th day of acclimation, followed by a gradual increase with the extension of acclimation time (Figure 5c). The water content continuously decreased with the extension of acclimation time, and significant differences were detected at 13 °C (*F* = 3.377, *p* < 0.05). However, no significant changes over time were observed at 21 °C (*F* = 1.726, *p* = 0.184) or 17 °C (*F* = 2.784, *p* = 0.055) (Figure 5d). Proline content showed an increasing trend with acclimation time, with significant differences at 13 °C (*F* = 2.994, *p* < 0.05). In contrast, no significant changes were detected over time at 21 °C (*F* = 0.973, *p* = 0.444) or 17 °C (*F* = 0.391, *p* = 0.812) (Figure 5e).

There were no significant differences compared with any of the control groups (25 °C). This demonstrates that none of the cryoprotectants in the control group exhibited statistically significant changes over time.

### 3.4. Changes in the Cold Tolerance of O. communa When Fed on A. artemisiifolia Under Different Treatments

After *O. communa* was fed on *A. artemisiifolia* subjected to different temperatures and durations, the physiological accumulations in the beetles changed accordingly.

**Figure 6:**
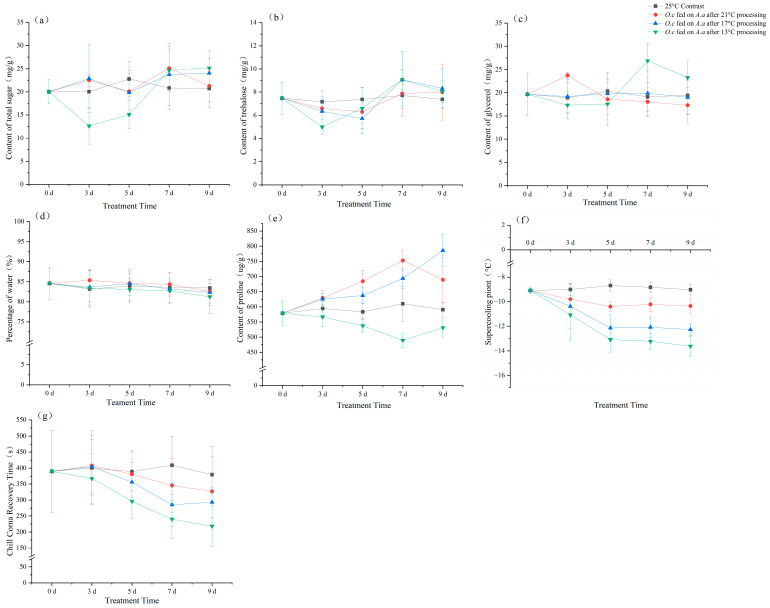
Physiological contents, the supercooling point (SCP) and chill coma recovery time (CCRT) of adult *O. communa* fed on cold-treated *A. artemisiifolia*. (**a**) total sugar content; (**b**) trehalose content; (**c**) glycerol content; (**d**) water content; (**e**) proline content; (**f**) SCP; (**g**) CCTR. The data are showed as mean ± SE.

For total sugar content, no significant temporal changes were observed in *O. communa* fed on *A. artemisiifolia* acclimated at 21 °C (*F* = 1.919, *p* = 0.147) or 17 °C (*F* = 0.746, *p* = 0.572). In contrast, the 13 °C treatment group showed a significant trend in total sugar content, characterized by an initial decrease followed by a marked increase, with the lowest value occurring in individuals fed on plants acclimated for 3 days. (*F* = 11.822, *p* < 0.05) (Figure 6a).

For trehalose content, no significant temporal changes were found in *O. communa* fed on *A. artemisiifolia* acclimated at 21 °C (*F* = 1.085, *p* = 0.39). In contrast, both the 17 °C (*F* = 3.364, *p* < 0.05) and 13 °C groups (*F* = 8.343, *p* < 0.05) showed a significant trend of initially decreasing and then increasing, with the maximum values observed after 7 days of plant acclimation. The minimum value in the 17 °C group occurred when insects were fed on plants acclimated for 5 days, whereas in the 13 °C group, the minimum occurred at 3 days (Figure 6b).

For glycerol content, no significant temporal changes were detected in the 17 °C group (*F* = 0.041, *p* = 0.997). In contrast, beetles fed on *A. artemisiifolia* acclimated at 21 °C (*F* = 3.271, *p* < 0.05) or 13 °C (*F* = 5.339, *p* < 0.05) showed a trend of first increasing and then decreasing, reaching its maximum at 3 or 5 days of plant acclimation (Figure 6c).

For water content, no significant differences were observed among all treatment groups (Figure 6d).

For proline content, beetles fed on plants acclimated at 21 °C showed a pattern of initial increase followed by a decrease, reaching the maximum at 7 days (*F* = 10.519, *p* < 0.05). In the 17 °C group, proline content gradually increased with acclimation duration (*F* = 24.378, *p* < 0.05). In contrast, in the 13 °C group, proline content first decreased and then increased, reaching a minimum at 7 days (*F* = 6.32, *p* < 0.05) (Figure 6e).

Regarding SCP, no significant temporal changes were observed in beetles fed on 21 –acclimated plants (*F* = 3.037, *p* = 0.055). However, when fed on plants acclimated at 17 °C (*F* = 9.466, *p* < 0.05) or 13 °C (*F* = 14.142, *p* < 0.05), SCP gradually decreased over time, indicating improved cold tolerance (Figure 6f).

Similarly, no significant temporal changes in CCRT were observed in beetles fed on plants acclimated at 21 °C (*F* = 0.562, *p* = 0.693) or 17 °C (*F* = 2.172, *p* = 0.109). In contrast, beetles fed on 13 –acclimated plants exhibited a significant decreasing trend in CCRT, reaching a minimum after 9 days, which further confirmed the enhancement of cold tolerance (*F* = 4.34, *p* < 0.05) (Figure 6g).

Overall, no significant differences were observed among the control groups.

## 4. Discussion

### 4.1. The Impact of Latitudinal Location on the Physiological Accumulates

When plants colonize new high-latitude environments, they initiate a series of physiological and molecular response mechanisms to mitigate damage caused by external low temperatures. Currently, it is recognizedthat physiological mechanisms are crucial for plants’ low-temperature responses, characterized by the massive synthesis of small-molecule substances such as free soluble sugars, polyols, and specific amino acids [21,23,24]. Cryoprotectants play multifunctional roles in enhancing cold tolerance beyond merely improving survival under lethal low temperatures. These compounds act as osmoprotectants to maintain cell osmotic balance, stabilize proteins and cell membrane structures, scavenge reactive oxygen species (ROS) induced by low-temperature stress, and regulate cellular metabolism to alleviate chilling injury. Such diverse functions are highly relevant to the physiological adaptation of insects and plants to low-temperature environments [25]. This defense mechanism enables invasive plants to gradually adapt to the complex and changeable local climate during their expansion process, which is why *A. artemisiifolia* has shown a tendency to spread to northern populations in China.

Similar to plants, insects also respond to low-temperature environments. Current research indicates that when insects enter autumn or winter, or are exposed to cold stress, they activate physiological regulatory mechanisms, primarily involving increased levels of cryoprotectants such as sugars, lipids, and proline [18,19]. Beyond these intrinsic mechanisms, host plants can also influence insect cold tolerance. This effect is largely attributed to differences in food quality and nutrient composition among host plants, which directly affect the insects’ nutritional intake and, consequently, the levels of cryoprotectants, enhancing their resistance to cold stress [20]. As ectothermic organisms, insects’ low-temperature adaptability is a key indicator of their environmental fitness. During the process of adapting to low temperatures and responding to cold stress, physiological changes in relevant metabolic substances play a pivotal role. The most widely recognized physiological mechanism underlying insect cold tolerance involves the accumulation of small-molecule cryoprotectants with high dry matter proportions and energy storage compounds. Numerous studies have confirmed the functional role of these cryoprotectants in insect cold tolerance, and research on cryoprotectants remains a focus in this field [26,27,28].

Through the determination of SCP and CCRT, we found that cold tolerance may be associated with the latitudinal location of the population. Consistent with the patterns of cold tolerance–related accumulates, carbohydrate-related substances in *A. artemisiifolia* and *O. communa* from the three higher-latitude regions were significantly higher than those in the Nanning population, exhibiting similar variation patterns between the two species. In contrast, glycerol content showed more variable changes: *A. artemisiifolia* from mid-latitude regions accumulated more glycerol, whereas *O. communa* from Nanning exhibited the highest glycerol content. This discrepancy may be attributed to a pronounced lag in glycerol accumulation, which is further amplified in *O. communa* through the feeding process compared to *A. artemisiifolia*. In this study, the increase in glycerol content in the 13 °C treatment group exhibited a clear lag phase, with no significant elevation observed until day 7. This lag can be explained by the time required for the activation of cold-responsive metabolic pathways: upon exposure to low temperature, the insect requires a period to upregulate the expression of key enzymes involved in glycerol biosynthesis (e.g., lipases and glycerol-3-phosphate dehydrogenase) and to achieve sufficient enzymatic activity to drive the accumulation of cryoprotectants. This delayed response also reflects an adaptive strategy, where glycerol synthesis is only triggered after prolonged cold exposure to avoid unnecessary energy expenditure under transient low temperatures. Regarding proline content, the Qinhuangdao population of *A. artemisiifolia* displayed the highest levels, while the Nanning population had the lowest. In *O. communa*, the Qinhuangdao population also had slightly higher proline content than other populations and showed stronger intraspecific stability, although no significant interspecific differences were detected. Although water is not a small-molecule cold tolerance substance, it can indirectly reflect the ratio of dry matter accumulation in organisms. In *A. artemisiifolia*, the water content of the two higher-latitude populations was significantly lower than that of the other two regions; in *O. communa*, the Nanning population had significantly lower water content than the other three populations, showing a general latitudinal trend. Firstly, northern populations of *A. artemisiifolia* and *O. communa* had a higher proportion of dry matter, including carbohydrates and lipids, which reflects their potential for cold tolerance adaptation. Secondly, lower water content in insects can greatly reduce the risk of intercellular ice formation, which is also an indicator of stronger cold tolerance [29].

Heritability reflects the capacity of parental traits to be transmitted to offspring, and it serves as a core indicator for evaluating the superiority or inferiority of different parental traits as well as the magnitude of heritability [30]. Based on the level of trait heritability, it can not only help determine the patterns of parental hybridization, the optimal time (generation) for offspring selection and the corresponding selection methods, but also predict the outcomes achieved under a specific selection intensity [31]. Previous studies on drought resistance identification of crops such as wheat and maize have demonstrated that the broad-sense heritability can be used as a reference indicator for genetic potential of drought resistance [32,33]. Our study found that both the cold tolerance-related phenotypes of *A. artemisiifolia* and *O. communa* exhibit high broad-sense heritability, demonstrating that genetic effects exert a greater influence on offspring trait formation than environmental factors. It is possible for the insect to overcome the cold climate in northern China either through further direct acclimation or by feeding on acclimated ragweed based on the results of this study. Although broad-sense heritability can serve as a reference for subsequent new variety breeding, and help us domesticate cold-tolerant leaf beetle populations with higher genetic stability for field release, it is less reliable than narrow-sense heritability.

### 4.2. The Physiological Accumulates of A. artemisiifolia Change with Acclimation Temperature and Duration

Temperature, as one of the key environmental factors influenced by latitude, significantly affects the growth, development, behavior, and survival of plants and insects [34]. Low temperatures delay plant growth and development, thereby impairing reproductive capacity, while insects exhibit reduced coordination and activity, increasing their risk of predation [35,36]. Plants from temperate regions of mid-low latitudes have been shown to develop enhanced cold tolerance after cold acclimation under non-freezing temperatures [37]. For insects, in addition to direct cold acclimation, host differences can also modulate their cold tolerance [38].

Low-temperature cold acclimation significantly improves the cold tolerance of *A. artemisiifolia*, primarily manifested by substantial increases in the contents of total sugars, trehalose, glycerol, and proline in treated plants. However, these substances exhibit distinct temporal dynamics under different acclimation temperatures. Total sugar content in *A. artemisiifolia* showed no significant difference from the control under Grade I acclimation, an overall increasing trend under Grade II, and an initial sharp decrease followed by a rapid rise under Grade III. Previous studies have reported that after cold acclimation, Camellia sinensis utilizes energy storage substances, such as carbohydrates, to enhance cold resistance, with starch being degraded to produce large amounts of sugars, including glucose, sucrose, and fructose. This process fluctuates significantly with time and temperature, reflecting the ratio between the rate of carbohydrate consumption and starch decomposition [39]. In our study, Grade I acclimation maintained this ratio at approximately 1; under Grade II, starch decomposition exceeded carbohydrate consumption; under Grade III, despite extensive starch decomposition, the rate was initially slower than carbohydrate consumption. Trehalose content in *A. artemisiifolia* showed little change compared to the control under Grade II, but increased significantly after 5 days under Grade I and III, which was similar to the dynamics of total sugar content. Although trehalose does not directly participate in energy metabolism in plants, it plays a crucial role in protecting biomolecular structures from damage and maintaining normal biological characteristics and life processes [40,41]. During the initial stage of cold acclimation when physiological activities are adequately supported, trehalose content remains dynamically stable; subsequent large-scale synthesis meets the plant’s demand for cold resistance, serving two primary purposes: directly lowering the cytoplasmic freezing point, and promoting abscisic acid (ABA) synthesis to induce protein production [42,43,44]. Glycerol content exhibited the most pronounced changes among all measured indicators, with significant differences detected under Grade I, II, and III cold acclimation treatments. In all groups, glycerol levels displayed a consistent pattern of an initial decrease followed by a subsequent increase. We speculate that this early dynamic pattern of glycerol is similar to that of total sugars. Under short-term or mild low-temperature stress, insects may preferentially allocate carbon resources toward the synthesis of energy-storage substances such as glycogen and lipids via gluconeogenesis and related biochemical pathways, rather than directly accumulating glycerol as a cryoprotectant. Only after prolonged or intensified cold exposure does the metabolic flux shift toward the production and gradual accumulation of glycerol, thereby enhancing cellular protection against low-temperature injury [45,46]. It is found that glycerol content in Musa acuminata increased with decreasing acclimation temperature, but this was not combined with temporal dynamics [47]. Proline content showed significant differences only under Grade III acclimation, with no obvious changes over time under Grade I and II. Reports have indicated that after cold acclimation, proline content in Triticum aestivum varies greatly with acclimation time and temperature, reaching the highest level after one week of acclimation at 12 °C [48]. This is consistent with the results of our study. As temperatures continue to decline, *A. artemisiifolia* accumulates proline more rapidly to maintain cellular osmotic balance and protect the stability of cells and macromolecules, thereby resisting more severe cold stress. Similar findings have been reported in *Carex*, *Duranta erecta*, and *Brugmansia arborea* [49,50].

### 4.3. The Impact of Differences in Host Physiological Accumulates on Insects

When *O. communa* fed on *A. artemisiifolia* acclimated under different temperature-time regimes, the contents of total sugars, trehalose, glycerol, and proline in the beetles increased within a certain range of conditions. Meanwhile, there was little change in water content, and both the SCP and CCRT were lower. These results indicate a significant enhancement in their cold tolerance, which is consistent with the experimental expectations. Since all treated beetles were reared under identical environmental conditions except for their food source, the increases in the contents of relevant metabolic and physiological substances and the enhanced cold tolerance are attributed to feeding on cold-acclimated *A. artemisiifolia*. In particular, under most conditions, the dynamic changes in the contents of total sugars, trehalose, glycerol, and proline were consistent between acclimated *A. artemisiifolia* and the beetles that fed on them. Therefore, it can be concluded that cold tolerance–related metabolites in *A. artemisiifolia* are transferred to *O. communa* through feeding and rapidly accumulate in the beetles via nutritional transfer, thereby enhancing their cold tolerance.

Currently, there are few reports on improving insect cold tolerance by cold-acclimating their hosts. Although numerous studies have confirmed that different hosts can lead to changes in insect cold tolerance, which is caused by nutritional differences, the underlying mechanism has not been clarified [18,19,20]. In recent years, research has found that physiological substances related to cold tolerance can be transferred through insect feeding behavior, and the nutritional transfer hypothesis for enhanced insect cold tolerance has been proposed [51]. For example, when exogenous proline was added to the diet of *Drosophila melanogaster*, tropical-origin flies showed a significant increase in internal proline content and cold tolerance after feeding, with their cold resistance strategy shifting from cold-sensitive to freeze-tolerant [52]. In addition, supplementing the host diet of *Nasonia vitripennis* with proline, alanine, or glycerol significantly improved their cold tolerance, with the proline-supplemented group showing the strongest effect. This result supports a nutrient-mediated transfer of cryoprotective compounds that contributes to cold tolerance. Furthermore, studies have shown that excessive concentrations of proline or alanine in the diet can reduce life-history parameters such as eclosion rate of *Sarcophaga crassipalpis* after feeding, which is detrimental to their survival, growth, and development [53]. The instability of such exogenous additives on predators also explains the phenomenon observed in this study where the content of certain cold-tolerance-related substances decreased rather than increased under specific temperature-time combinations. This “instability” refers to the inconsistent absorption, metabolism, and retention of exogenous cryoprotective substances in predatory insects. However, under the combined effects of various substances, *O. communa* still showed enhanced cold tolerance.

## 5. Conclusions

To survive in particular habitats, insects generally require the capacity to tolerate cold environmental conditions [54]. Cold acclimation, heritability, and evolutionary potential are major determinants that shape an organism’s cold tolerance [55]. Cold acclimation has been found to confer substantial benefits at low temperatures; thus, only acclimated flies were able to locate resources when released into cold environments [56]. Since insect pest predators tend to follow their prey into new areas, cold tolerance constitutes a critical component of their survival toolkit [57,58]. *A. artemisiifolia* exhibits a south-to-north invasive pattern across China. For effective population control of this ragweed, the herbivorous beetle *O. communa* must evolve adaptive capacity to withstand low temperatures in winter.

In conclusion, low temperatures affect the physiological status of plants, and feeding by insect herbivores further alters these physiological levels in insects, enhancing their cold tolerance. This indicates the trophic transmission of cold tolerance between plants and insect herbivores. Therefore, insects are suggested to accumulate cryoprotectants either through their own metabolism or via feeding on host plants to resist cold stress. This mechanism can assist in the cultivation of natural enemy insects with high adaptability to address the growing problem of plant ecological invasion. In subsequent studies, we plan to re-examine field-collected beetles to obtain the cold tolerance phenotypic indices of male and female individuals across three consecutive generations. These data will be used for the analysis of narrow-sense heritability, allowing the identification of optimal generation for the selection of cold-tolerant individuals. Future research should also focus on the molecular mechanisms underlying cryoprotectant transmission and long-term fitness of cold-adapted insects under field conditions.

## Figures and Tables

**Table 1:** Broad-sense heritability (H^2^), variance, and coefficient of variation components for the physiological indicators of *A. artemisiifolia* and *O. communa* beetles.

	*Ambrosia artemisiifolia*					*Ophraella communa*						
	Total Sugar	Trehalose	Glycerol	Water	Proline	Total Sugar	Trehalose	Glycerol	Water	Proline	SCP	CCRT
Mean ± SE	7.16 ± 1.07	2.09 ± 0.34	5.04 ± 0.82	0.75 ± 0.02	60.02 ± 6.04	15.96 ± 3.12	13.98 ± 2.97	13.61 ± 2.63	0.8149 ± 0.0397	583.57 ± 57.96	−10.93 ± 0.58	142 ± 49.48
V_G_	17.085	1.7646	5.909	0.006191	762.7	135.62	166.36	31.64	0.01797	3019	6.529	20,829
V_E_	1.396	0.1251	0.714	0.000431	38.7	12.27	11.88	8.09	0.001746	3585	0.343	2576
V_P_	17.3642	1.78902	6.0518	0.0062772	774.44	138.074	168.736	33.262	0.018319	3736	6.5976	21,151
H^2^	0.984	0.986	0.976	0.986	0.989	0.982	0.986	0.951	0.981	0.808	0.99	0.985
N	5	5	5	5	5	5	5	5	5	5	8	8

Genetic variance (V_G_), environmental variance (V_E_), phenotypic variance (V_P_), broad-sense heritability (H^2^) for physiological indicators. N = sample size.

## Data Availability

The datasets used and analyzed in this study are available from the corresponding authors upon request.
